# Impacts of agricultural expansion on the resource availability of forest-dependent Indigenous communities in the Dry Chaco

**DOI:** 10.1007/s13280-025-02217-6

**Published:** 2025-07-31

**Authors:** María Vallejos, Ana Álvarez, Olivia del Giorgio, Tobias Kuemmerle

**Affiliations:** 1 Geography Department, Humboldt-Universität zu Berlin, Unter den Linden 6, 10099 Berlin, Germany; 2 Instituto de Investigaciones Fisiológicas y Ecológicas Vinculadas a la Agricultura (IFEVA), Universidad de Buenos Aires, CONICET, Buenos Aires, Argentina; 3 Departamento de Métodos Cuantitativos y Sistemas de Información, Facultad de Agronomía, Universidad de Buenos Aires, Buenos Aires, Argentina; 4 Acompañamiento Social de la Iglesia Anglicana en el Norte Argentino (ASOCIANA), General Güemes 1180, 4400 Salta, Argentina; 5 Department of Geography, McGill University, Burnside Hall Building, 805 Sherbrooke Street West, Montreal, QC H3A 0B9 Canada; 6 Integrative Research Institute on Transformations of Human-Environment Systems (IRI THESys), Humboldt-Universität zu Berlin, Berlin, Germany

**Keywords:** Agricultural frontiers, Forest-dependent people, Inequities, Natural resource access, Tropical dry woodlands and savannas, Vulnerability

## Abstract

Agricultural expansion into tropical and subtropical forests threatens forest-dependent communities by disrupting their access to vital resources. We explored these impacts for over 400 Indigenous communities in the Argentine Dry Chaco, a deforestation hotspot due to agricultural expansion. Using participatory mapping, we estimated resource collection footprints for plants and animals, and integrated these with deforestation data mapped from satellite images to show that by 2021, communities had lost on average 21% of their forests. An ecosystem services supply index revealed that 33% of communities saw 10–35% reductions in resource availability in 2001–2021. We also found substantial increase in access restrictions (42%), and communities had to travel over 10 km further to reach natural water sources. These findings highlight the severe consequences of agricultural expansion on Indigenous communities in the Chaco and likely many other dry forest regions, emphasizing the need for policies to prevent ecological marginalization of forest-dependent communities.

**Supplementary Information:**

The online version contains supplementary material available at 10.1007/s13280-025-02217-6.

## Introduction

Tropical and subtropical forests provide a diverse stream of benefits at different scales for billions of people worldwide (Shackleton and de Vos 2022). For communities living inside or near these forests, access to such resources serves as a critical safety net (Newton et al. 2016). The continued expansion of industrialized agriculture in many tropical countries threatens to disrupt the delivery and accessibility of forest-based resources, particularly in regions already grappling with high levels of vulnerability (Laurance et al. 2014). This disruption can jeopardize local livelihoods, exacerbate conflicts, and drive rural outmigration (Cáceres 2015; Scheidel et al. 2023). Despite these potential impacts, there is a notable gap in our understanding of the effects of land-use changes on forest-dependent communities, translating into barriers to policy and planning seeking to achieve sustainable development. In particular, spatial data on the potential declines in forest-resource availability and accessibility can highlight the challenges faced by forest-dependent groups, allowing to ‘put them on the map’ and to support targeted prevention and protection measures to enhance their quality of life (Garnett et al. 2018; Levers et al. 2021). However, approaches for capturing the spatial footprint of resource use of local communities are often missing.

Indigenous Peoples around the world often depend on forests for their cultural, economic, and social well-being (Newton et al. 2016). Forests not only provide resources such as food, medicinal plants, and raw materials for construction and handicrafts for these communities, but also hold profound cultural and spiritual significance (Sangha et al. 2015). In 2022, a milestone in global conservation efforts was achieved by adopting the Kunming-Montreal Global Biodiversity Framework, which sets ambitious, actionable targets to curb biodiversity loss by 2030. This framework not only aims to safeguard the planet's biodiversity but also emphasizes the critical role of Indigenous Peoples and local communnvironmental contaminatioities (Fa et al. 2020). It explicitly calls on nations to recognize and protect the rights of these groups, whose traditional knowledge and stewardship are essential for effective conservation. However, despite this recognition, the enforcement of Indigenous rights remains weak, with many communities facing persistent challenges in securing land tenure and access to resources (United Nations 2021).

Dry forests cover approximately 42% of the tropical and subtropical forested areas and play a crucial role in supporting the livelihoods of forest-dependent communities (Hasnat and Hossain 2020). These forests, characterized by unique biodiversity, provide essential forest-based resources (Miles et al. 2006). However, many dry forests face high and increasing pressures from expanding agriculture, logging, and other land use, often more so than rainforests (Blackie et al. 2014; Buchadas et al. 2022). Despite the importance of dry forests, the impacts of the expansion of industrialized agriculture and the resulting forest loss in these regions on local communities remain understudied (Sánchez-Azofeifa et al. 2005). This translates into high uncertainty regarding the vulnerability of people living in dry forests.

Agricultural expansion into dry forests reduces biomass and accelerates the degradation of remaining forests. This, in turn, can alter the availability and accessibility of natural resources for forest-dependent communities, threatening their livelihoods. To understand the magnitude of these impacts, it is necessary to define the areas of forest use of these communities and to estimate changes in resource availability within those areas. The "cascade" model of ecosystem services (Haines-Young and Potschin 2009; de Groot et al. 2010) conceptualizes the flow from ecosystem structure and processes through functions to services, which generate benefits that ultimately hold value for human well-being. Using this approach, it is possible to link ecosystem structure and functioning (such as carbon sequestration or water regulation) with the direct benefits that local populations obtain from ecosystems (i.e., provisioning ecosystem services). Participatory mapping can provide valuable information in this context by defining the areas of use of forest-based resources (such as wild food, freshwater, fuelwood, or medicinal plants) through the participation of key community members who provide information and knowledge about the spatial use of forests (Ramirez-Gomez et al. 2016). Moreover, by estimating changes in key functional aspects of ecosystems that underpin provisioning services in these areas, it is possible to detect fluctuations in resource availability for local populations (Paruelo et al. 2016; Barral et al. 2020). However, these combined approaches have rarely been applied in tropical dry forests.

In addition to the loss of forest due to conversion to some other land use, where agricultural expansion takes place, access to remaining forests becomes increasingly restricted due to the claiming of land and the construction of physical barriers such as fences by agribusiness actors (del Giorgio et al. 2021, 2025). This further limits resource access and can exacerbate local communities’ vulnerability. A key resource in this context in dry forests is access to water, as land claiming and deforestation can lead to changes in the availability, distribution and access to water sources (IPCC 2023). This poses major risks for communities, underscoring the need for comprehensive assessments of access to forest and water resources to formulate effective strategies for protecting these communities in situations where they often face major power imbalances.

In this paper, we developed an approach to evaluate the impact of the expansion of industrialized agriculture on local Indigenous communities due to changes in the availability of forest-based resources and the accessibility of these resources. We demonstrate this approach for the eastern portion of the province of Salta in the Argentinian Dry Chaco, a global deforestation hotspot due to agricultural expansion and a region of uniquely high ethnic diversity (Casimiro Córdoba 2019; Buchadas et al. 2022). Specifically, we asked the following research questions:

1. What is the spatial footprint of forest use by Indigenous communities, and how has forest loss impacted these areas between 2001 and 2021?
2. How has the availability of forest-based resources in areas used by Indigenous communities changed from 2001 to 2021?
3. To what extent has access to natural resources for Indigenous communities been altered due to agricultural expansion during the same period?

We addressed these questions with the aim of help making visible the so far largely hidden impacts of the region’s agriculturization on Indigenous communities in the region, using a combination of participatory mapping and remotely-sensed indicators of availability and access to forest-based resources.

## Materials and methods

### Study area

Salta is a province in northern Argentina, with its eastern region situated in the South American Dry Chaco. The region is semiarid, characterized by a strong seasonality, with humid, hot summers and dry winters (Berbery and Barros 2002). Its vegetation comprises semi-deciduous xerophytic forests interspersed with shrublands, savannas, and grasslands (Morello et al. 2012). Recognized as a biodiversity hotspot, this region is home to over 400 bird species, 150 mammals, 120 reptiles, and 100 amphibians (The Nature Conservancy 2005). Since the turn of the century, Chaco has become a global deforestation hotspot (Kuemmerle et al. 2017), with the dominant drivers of forest-clearing being the expansion of industrialized agriculture, primarily soybean and maize for export, and cattle ranching for domestic beef consumption (Baumann et al. 2022).

**Fig. 1:**
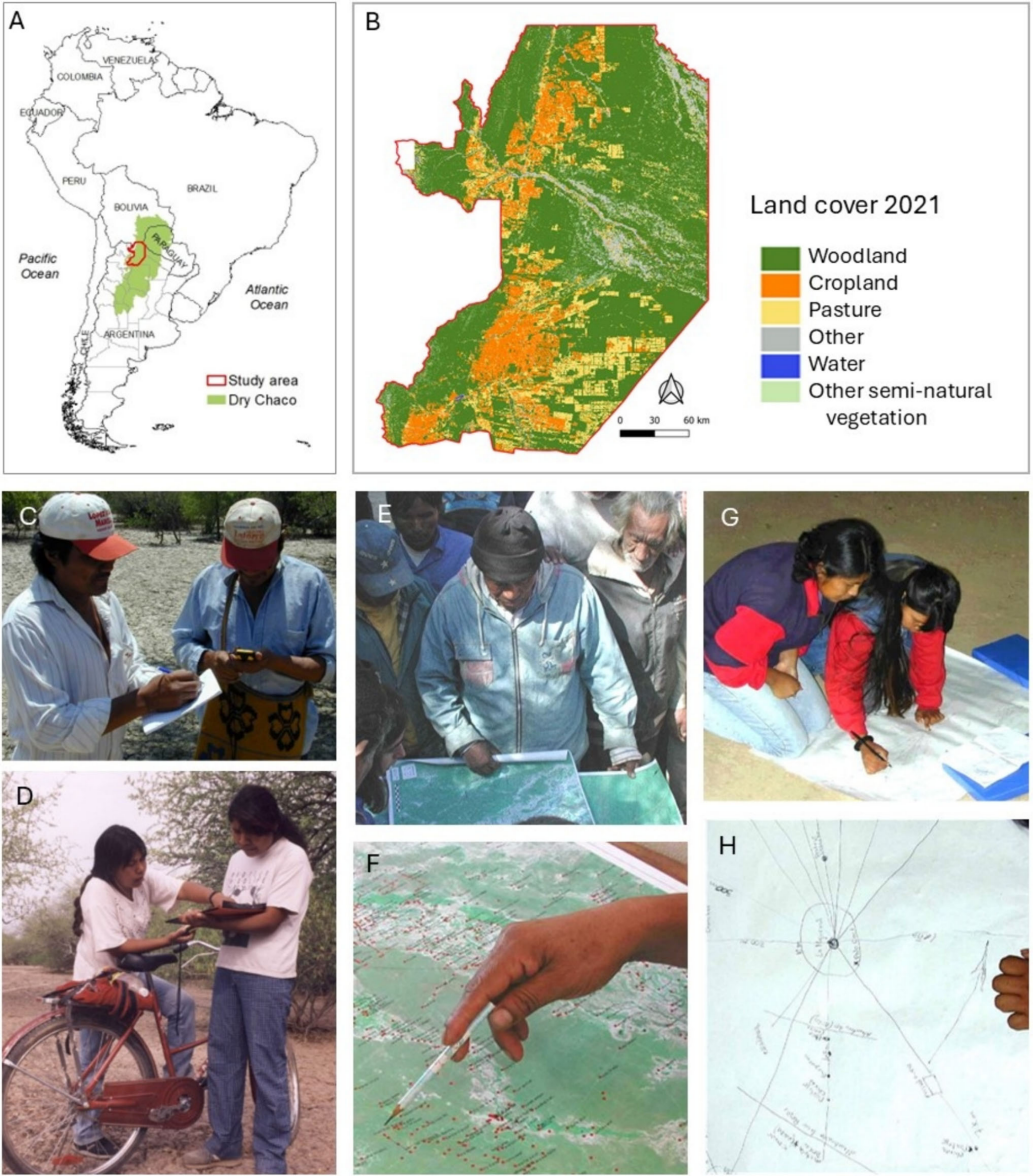
Participatory mapping to locate the areas of collection of forest resources with the Indigenous communities of eastern Salta, which we subsequently used to estimate the spatial footprints of forest use. **A**. Study site – eastern Salta, Argentina; **B**. Land use/cover in eastern Salta in 2021 (Baumann et al. 2022); **C**, **D**. Demarcation of collection sites by communities using GPS, **E**, **F**. by using printed maps to pinpoint sites, and G-H by means of graphical representation using sketches (Photos: ASOCIANA)

Within the Argentine Chaco, Salta is one of the provinces with the highest deforestation rates (Vallejos et al. 2015). By the end of 2021, 2187 million hectares (Mha) of forest had been converted to cropland or pastures (27% loss) across the five counties (or *departamentos*): Anta, Metán, Orán, Rivadavia, and San Martín (Fig. 1A, B; Figure S1; Baumann et al. 2022). Simultaneously, Salta harbors the greatest ethnic diversity in Argentina, including Indigenous Peoples belonging to the Guaraní, Wichí, Kolla and Diaguitas groups, and to a lesser extent, Chanés, Chorotes, Calchaquíes, Tobas, Tapietes, Chulupíes, Lules, Atacamas and Tastiles groups (INDEC 2022). According to the last National Population Census, 10% of the population of Salta identified as Indigenous in 2022 (142,870 persons in total), more than triple the Argentinean average (2.9%) (INDEC 2022). Of the total number of Indigenous communities in the eastern part of Salta only 17% had land ownership titles at the beginning of the 2000s, and 65% of them were making territorial claims (Leake 2008). Alongside having among the highest poverty rates in the country (Longhi et al. 2020), the region also has high levels of tenure insecurity (Collins et al. 2024).

The Indigenous Peoples of this region traditionally led nomadic lifestyles as hunters, gatherers, and fishers (Palmer 2005). While many now live in permanent settlements and some have integrated into the labour market, the majority still rely on the forest for their subsistence. For Indigenous communities, the relationship with the land is not merely a matter of possession and production but must be recognized and understood as the fundamental basis of their cultures, spiritual life, and integrity (CIDH 2009). Their territories and resources constitute, in addition to their main means of subsistence, an integral element of their worldview, religiosity and, therefore, of their cultural identity (CIDH 2009). From an Indigenous perspective, the forest is seen as a rich source of resources, providing timber for building homes, wild fruits from trees, animals for hunting, medicinal plants, and materials for artisanal production and dyes (Leake 2008). According to the Chané Indigenous worldview, for example, the physical space where their different activities take place is conceived in circles, each of which refers to the family (*“oka”*), the community (*“tëta”*), the arable land (*“koo”*) and the forest (*“kaa”*). The *“kaa”* is a wide and extensive area that represents a vital space of nature, where animals, trees, medicinal herbs, rivers and other plants are found (Hirsch et al. 2016). According to the Wichí Indigenous worldview, each natural resource has its “owner” or “lord” who must be asked for permission to hunt or fish. In this way, the sustainability of the resources for common use is ensured through rational-use guidelines (Suárez 2012). For this reason, private-property logic does not correspond well to the Indigenous concept of communal space, where members of the community have equal access to the land and natural resources, with access and usage rights governed by social norms, customs, or collective agreements.

Indigenous communities possess extensive knowledge of the local geography, as well as the region's flora and fauna. For instance, an ethnobotanical study found that the Indigenous communities of eastern Salta use a very wide range of species, including 75 plant species, 47 mammal species, 78 bird species, 14 reptile species, 3 amphibian species, 19 species of bees and wasps, and 3 other insect species (Arenas 2003). Likewise, the fruits of Algarrobo (*Neltuma alba*), Mistol (*Sarcomphalus mistol*), Tusca (*Vachellia aroma*), and Chañar (*Geoffroea decorticans*) are collected for their high nutritional content (Menna and Bianco 2015). In addition, armadillos (e.g., Quirquincho, Pichi), Chacoan peccaries (*Catagonus wagneri*), Chacoan mara (*Pediolargus salinicola*), grey brocket deer (*Mazama guszobira*), Chaco chachalacas (*Ortalis canicollis*), and tegu lizards (*Tupinambis* spp.) are hunted for meat (Camino et al. 2018). According to another ethnobotanical study, Wichí communities rely on 115 plant species for medicinal purposes, using them to treat ailments such as digestive and respiratory issues, as well as menstrual pain (Suárez 2019). Additionally, the "chaguar" (*Bromelia hieronymi*) serves as a fibre source for spinning and weaving, while also holding deep cultural significance within these communities (Van Dam 2000; Montani 2008).

### Spatial footprint of forest use

To define the location of Indigenous settlements in the study area, we used two sources of information: a map of native peoples in Argentina, developed by the National Institute for Indigenous Affairs (INAI, Argentina); and a database of Indigenous Peoples of the 'Chaco Salteño' (Leake 2008). These sources include the location of 349 and 202 communities, respectively. We overlayed both sources to eliminate duplicate records. As our focus was on forest-based resource use, we discarded communities that coincided with urban areas (assuming that these communities are not forest-dependent). This resulted in a total of 444 Indigenous communities that were included in our analysis. The location of the Indigenous settlements in the study area is spatially uneven—231 of the communities are located in the county San Martín (52%), 156 in Rivadavia (35%), 35 in Orán (8%), in Anta 19 (4%) and 3 in Metán (1%)—and ethnically diverse—165 communities (37%) recognizing themselves as *Wichi*, 48 as *Guaraní* (11%), 21 as *Kolla* (5%), 16 as *Chorote* (4%), 13 as *Ava Guarani* (3%), 10 as *Qom* (2%), 9 as *Logys* (2%), 5 as *Tupi Guaraní* (1%), 4 as *Chan* (1%), 4 as Chiriguanos, Chulupí, Calchaquí, Lule Viela ethnic groups (1% altogether), and for the remaining 33% we had no data on ethnic self-identification.

To estimate the area of use for the different activities carried out by Indigenous communities, we relied on data from a participatory mapping project conducted between 1998 and 2002. This project was initiated and coordinated by ASOCIANA, a local NGO, in collaboration with the National University of Salta (UNSa), and Indigenous leaders (data summarized by Leake 2008). The project engaged 71 Indigenous communities across the study area (42 in Rivadavia, 26 in San Martín and 3 in Anta), aiming to estimate the distances travelled by community members to gather various natural resources. Indigenous communities hold authorship rights over the data generated in this study. Nine resource-related activities were selected—hunting, fishing, fruit gathering, honey harvesting, firewood cutting, charcoal production, house construction, carpentry, and handicraft making—representing activities regularly performed by men and/or women. Structured interviews were used to identify collection sites of significant value to Indigenous communities. Each survey began with a community meeting, inviting all members to introduce the project's objectives and methodology. Interviewers, together with Indigenous assistants, then visited each household to conduct in-depth interviews. GPS devices were provided to Indigenous representatives, and workshops were conducted to train them in recording the coordinates of collection sites. In parallel, additional meetings were held with community members—both men and women—using pencils and markers to pinpoint collection locations on large printed maps based on Landsat satellite images, as well as through sketches and graphical representations, which were then translated into the map (Fig. 1C–H).

To delimit the spatial footprints of resource use of Indigenous communities, we estimated the distances from each surveyed Indigenous settlement to the sites of collection in a GIS (QGIS Development Team, 2023). We classified resources into two types, plant- and animal-based, due to the different nature of both groups of resources (animals move in space, while plants do not, which influences the type of collection or hunting), and used these classifications to define buffers around each community. The buffer size was determined by the median distance to the farthest resource collected per type, ensuring that all resources within each category were encompassed.

To estimate the forest loss in the spatial footprint of resource use between 2001 and 2021, we overlapped the spatial footprint of resource use around each of the 444 community settlements with the area that was deforested in this period. We obtained deforested data from a plot-level, hand-digitized geo-database of deforestation due to agricultural expansion, generated based on Landsat satellite images^1^. The mean plot size in eastern Salta according to this database is 77.0 ± 1.4 ha. To assess whether differences in deforestation areas within footprints were significant between both years, we checked normality of the data distribution, homogeneity of the variances, and used paired t-tests (in the case of confirmed normal distributions) or the non-parametric Wilcoxon Signed-Rank test (for non-normal distributions).

### Changes in the availability of natural resources

To estimate changes in the availability of natural resources in the spatial footprint of resource use, we applied the Ecosystem Services Supply Index (ESSI), a proxy of ecosystem functionality that has shown to correlate well with the availability of supporting ecosystem services, including carbon and water dynamics, soil organic carbon, and biodiversity (Paruelo et al. 2016). This index is a compound measure of ecosystem functionality and is composed of two attributes of the seasonal dynamics of the Normalized Difference Vegetation Index (NDVI): the annual mean (NDVI_mean_)–an indicator of the total productivity and carbon gains–and the intra-annual Coefficient of Variation of the NDVI (NDVI_CV_)–a descriptor of the seasonality of those carbon gains; where ESSI = NDVI_mean_ *(1-NDVI_CV_).

To calculate ESSI mean for each spatial footprint of resource use, we used the NDVI product based on MODIS sensor data, due to its high temporal resolution, which allows for better capture of vegetation phenology. We used quality flags to exclude pixels affected by clouds, cloud shadows, and aerosols. We then standardized ESSI values by normalizing them to the 0–1 range. ESSI values close to 1 indicate both a high productivity and a high temporal stability (or lower seasonality) of productivity and are related to a higher supply of ecosystem services. We calculated the annual value of ESSI pixel by pixel for 2001 and 2021 for the study area and calculated mean ESSI values for each spatial footprint of resource use. We used Google Earth Engine (Gorelick et al. 2017) to perform this analysis. To assess whether differences in the ESSI mean values within footprints were significant between both years, we checked normality of the data distribution, homogeneity of the variances, and used paired t-tests or Wilcoxon Signed-Rank test to assess differences.

### Changes in access to natural resources

To capture changes in the access to forest-based resources, we used a database of forest demarcation lines produced by del Giorgio et al. (2025). These linear deforested strips represent land claims, oftentimes made by agribusiness and land speculators, and are frequently accompanied by fences (adding legal weight to the claim and serving as a deterrent for trespassing) (del Giorgio et al. 2021). These demarcations overlap with lands traditionally used by Indigenous communities, who for the most part do not have legal tenure. The demarcation database was constructed via the detection of linear features, using Sentinel-2 imagery (10 m resolution) for 2020, which were then cleaned (i.e., masking roads, rivers and other line segments that are not demarcation lines). Using trend analysis, each linear segment was then assigned a year of appearance, based on Landsat image time series analysis from 1986 to 2020. For our analysis, we calculated the density of forest demarcations within each spatial footprint of resource use to approximate the intensity of land claiming and potential concomitant access restrictions. We then calculated the weighted density of demarcation lines by putting into relation the total density of demarcations and the forest area available within each spatial footprint of resource use in 2020. To assess the change in claiming intensity, we calculated density measures for both 2001 and 2020. It should be noted that, because the forest demarcations were mapped for 2020, our 2001 density measures do not account for demarcations made within plots converted to agriculture by 2020. It should also be noted that several communities (n = 15, 4%) in the west of the study area, whose footprint of use was not fully encompassed by the demarcation dataset, were excluded from this part of the analysis. To assess whether differences in the demarcation density within footprints were significant between both years, we tested for differences as described above.

To estimate access to water resources, we used the JRC Global Surface Water Mapping Layers, v1.4 (Pekel et al. 2016). This dataset was generated from Landsat satellite images and maps the spatial–temporal distribution of surface water within each year for the period 1984 to 2021, at 30 m resolution. Based on these data, we extracted water bodies using the 'max_extent’ band, and used the 'waterClass' band, which gives information about the seasonality of water throughout the year, to classify water bodies into permanent or seasonal. Given the extended dry period in the study region, access to permanent water is particularly critical during the dry season. We also stratified all permanent water bodies into rivers and reservoirs by visual interpretation. Furthermore, we located urban areas as a possible alternative source of drinkable water (under the assumption that cities have running water that can be accessed by community members). We estimated the distance that each community must travel to access water in 2021, by calculating the distance to the nearest water source – seasonal or permanent water bodies plus urban areas. Finally, we estimated changes in the availability of permanent water between 2001 and 2021.

## Results

### Spatial footprint of forest use and forest loss within this area

**Fig. 2:**
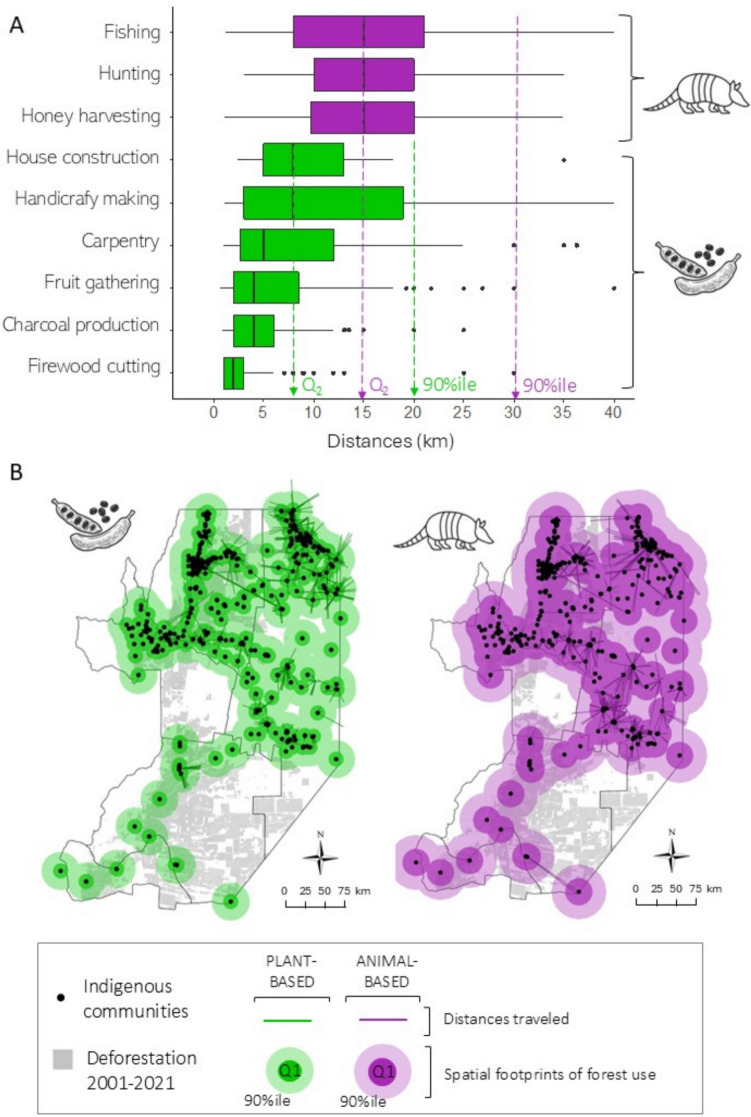
Estimated spatial footprints of forest use. **A**. Distances travelled to collect natural resources for different activities surveyed, which were obtained with the participatory mapping; **B**. Estimated spatial footprint for plant- and animal-based resources, by using the median (Q_2_) of the distances travelled for resource collection of both types

The participatory mapping identified a total of 1458 collection sites, of which 808 sites were for plant resources and 650 for animal-based resources. In general, we observed that communities travelled greater distances to capture animal-based compared to plant-based resources. The median distance for collecting plant-based resources varied between 2 km (for firewood) and 8 km (for house construction and handicraft making), while the median distance travelled for the collection of animal-based resources was 15 km across all three animal-based resources (Fig. 2A, Figure S2). Given these results, we defined the plant-based spatial footprint as an 8 km buffer around communities, covering a total area of 17,301 km^2^ for all communities. For the animal-based spatial footprint, we used a 15 km buffer around communities, resulting in a cumulative area footprint of animal-based collection of 46 528 km^2^ (Fig. 2B).

**Fig. 3:**
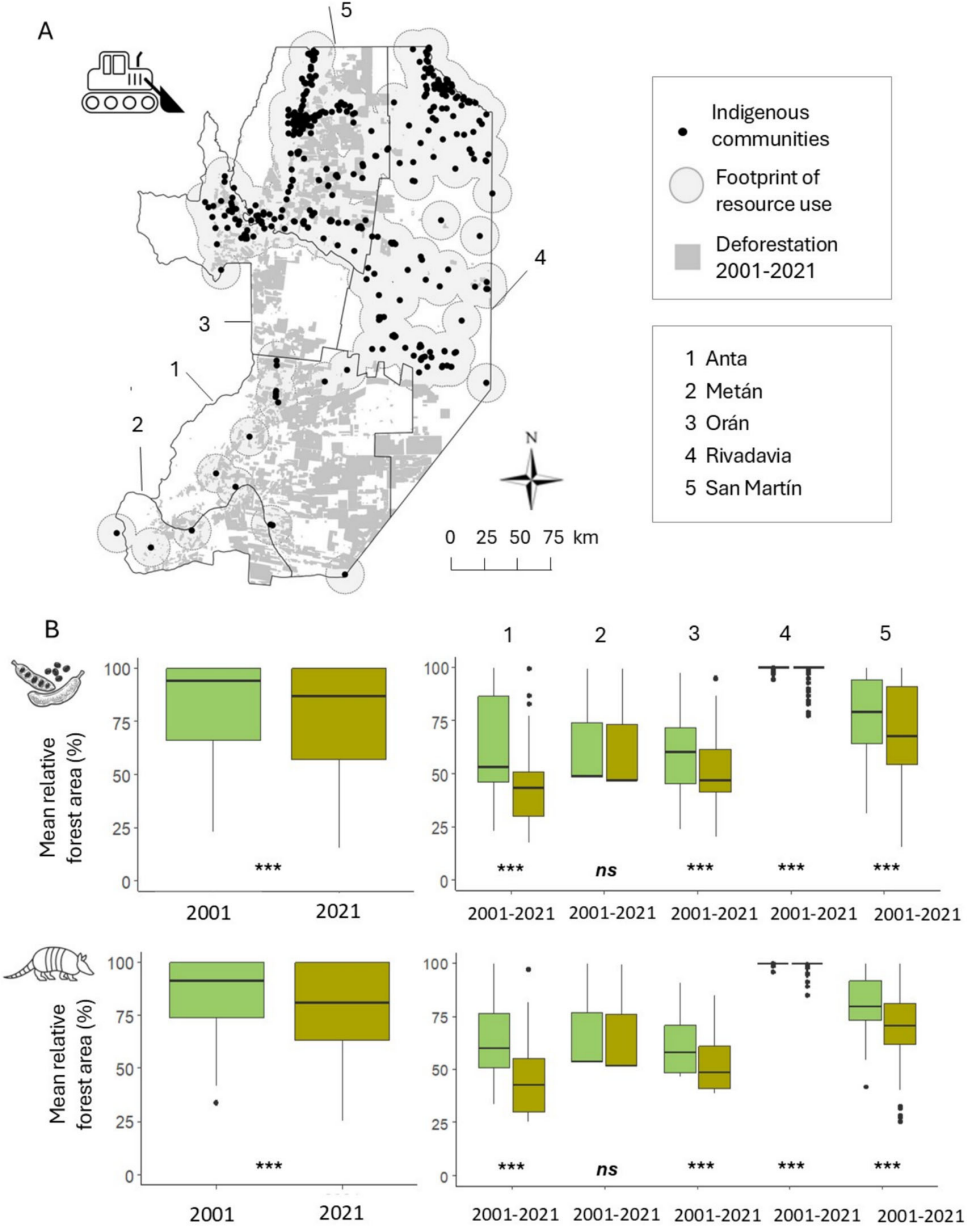
Forest loss within the spatial footprints of forest use between 2001 and 2021. A. Map of the deforested area between 2001 and 2021 in the study area; B. Mean relative forest area (%) in the spatial footprints for plant-based (8 km buffer; top) and animal-based (15 km; bottom) resources in 2001 and 2021, for the whole study area (left) and by county (right). Significance levels: ***(*p* < 0.001); **(*p* < 0.01); *(*p* < 0.05); ns (not significant)

Comparing the spatial footprints of forest use to the patterns of forest loss between 2001 and 2021 showed that a total of 4329 km^2^ of forest had been converted to agriculture in that period inside the spatial footprint of resource use, representing 9% of forest loss within this area. This, combined with the 4214 km^2^ already converted by 2001, amounts to a total area of conversion of 8543 km^2^. In 2021, the average forest loss across communities inside the spatial footprint of resource use was 4510.6 ± 419.6 ha, representing a relative loss of 21.4 ± 2.0%. A total of 68 communities (15% of all communities) had lost more than 50% of their resource base area. Relative forest loss between 2001 and 2021 was greater in the areas used for collecting animal-based resources (6.5 ± 0.7%) than in the areas used for collecting plant-based resources (5.3 ± 0.7%) (Fig. 3; Table S1A, B).

Forest loss was geographically highly unevenly distributed across the resource-use footprints. Indigenous communities living in Anta experienced the most substantial reductions in forest area between 2001 and 2021, with significant relative losses of 14.6 ± 5.9% in plant-based resource footprints and 18.5 ± 3.4% in animal-based resource footprints *(all p* < *0.001)*. In contrast, communities in San Martín and Orán experienced moderate forest losses, with significant relative reductions ranging from 7.3 ± 1.0 to 9.1 ± 0.8% on average for plant- and animal-based resource footprints *(all p* < *0.001)*, respectively. Indigenous communities in Metán and Rivadavia were the least affected, with relative forest area losses of 0.9 ± 0.5% *(not significant)* and 0.7 ± 0.3% *(p* < *0.001)* on average for plant and animal-based resource footprints, respectively (Fig. 3; Table S5 A–C).

### Changes in the availability of natural resources

**Fig. 4:**
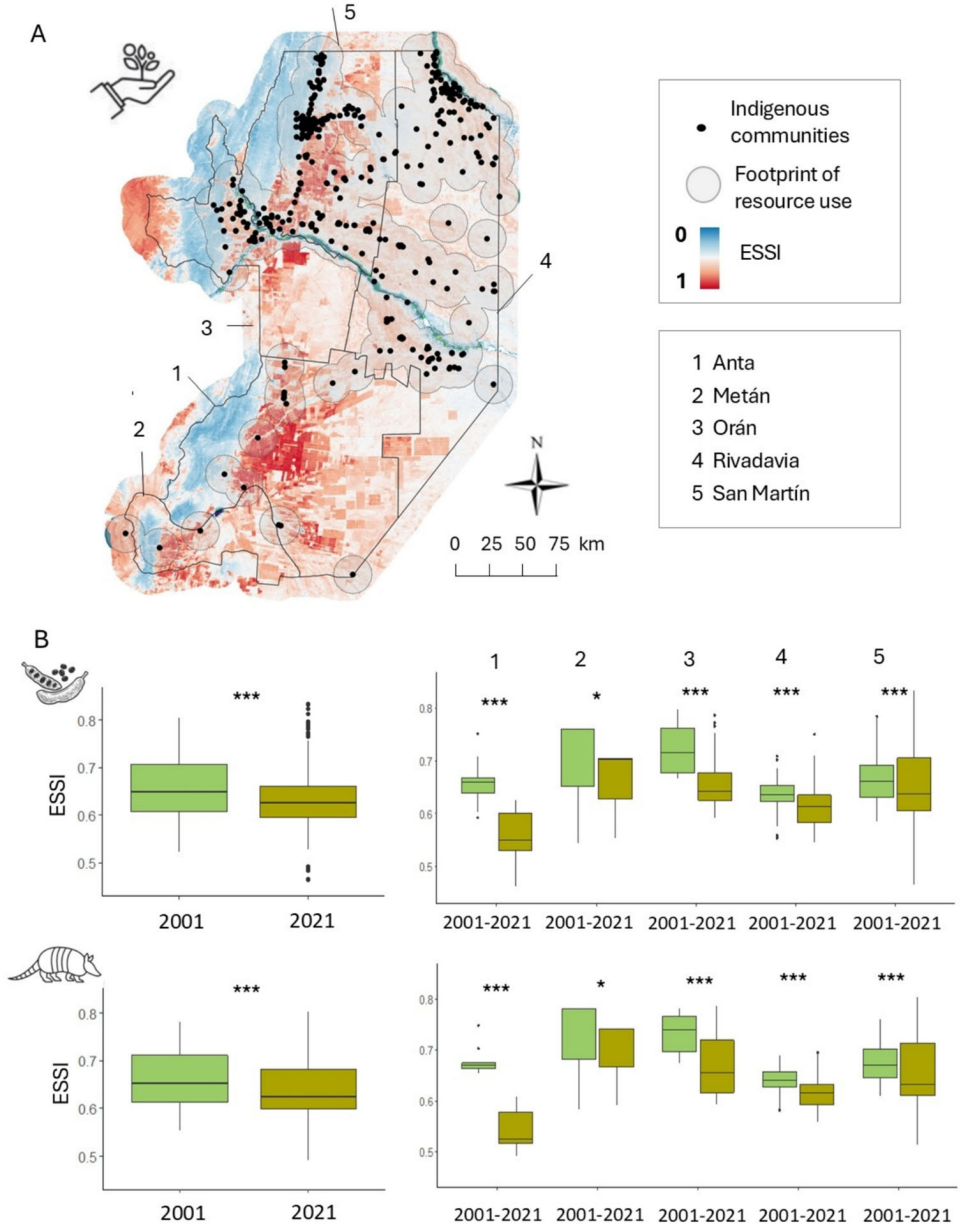
Ecosystem service provisioning within the spatial footprints of forest use in 2001 and 2021, based on the Ecosystem Services Supply Index (ESSI). **A**. Map of ESSI values in the study area by. **B**. ESSI values in the spatial footprints for plant-based (8 km buffer; top) and animal-based (15 km; bottom) resources in 2001 and 2021, for the whole study area (left) and by county (right). Significance levels: ***(*p* < 0.001); **(*p* < 0.01); *p < 0.05); ns (not significant)

In 2021, Anta had the lowest absolute values of the Ecosystem Services Supply Index (ESSI)—a proxy for natural resource availability—within the spatial footprints of forest use, providing 15% fewer ecosystem services than the average ESSI values across all footprints. Rivadavia also exhibited low ESSI values, with levels 4% below the general average (Table S2). By 2021, 148 communities (33% of the total) experienced a reduction between 10 and 35% in natural resource availability—measured as ecosystem services supply—within the spatial footprints of forest use compared to 2001. On the contrary, 36 communities (9%) experienced an increase of 6–9% in natural resource availability compared to 2001. Comparing ESSI values from 2001 to 2021, we observed slight but significant average declines of 3.8 ± 0.3% for plant-based resources and 4.3 ± 0.3% for animal-based resources. Analysing our results by county, we found stark differences between them. The most affected county was Anta, with a marked decline in the ESSI between 2001 and 2021 —on average for plant and animal-based resources—by 17.5 ± 1.2% *(p* < *0.001)*, followed by Orán (8.5 ± 0.6%), Rivadavia (3.7 ± 0.3%), Metán (3.6 ± 1.7%), and San Martín (2.5 ± 0.3%) *(all p* < *0.001)* (Fig. 4; Table S6 A–C).

### Changes in access to natural resources

**Fig. 5:**
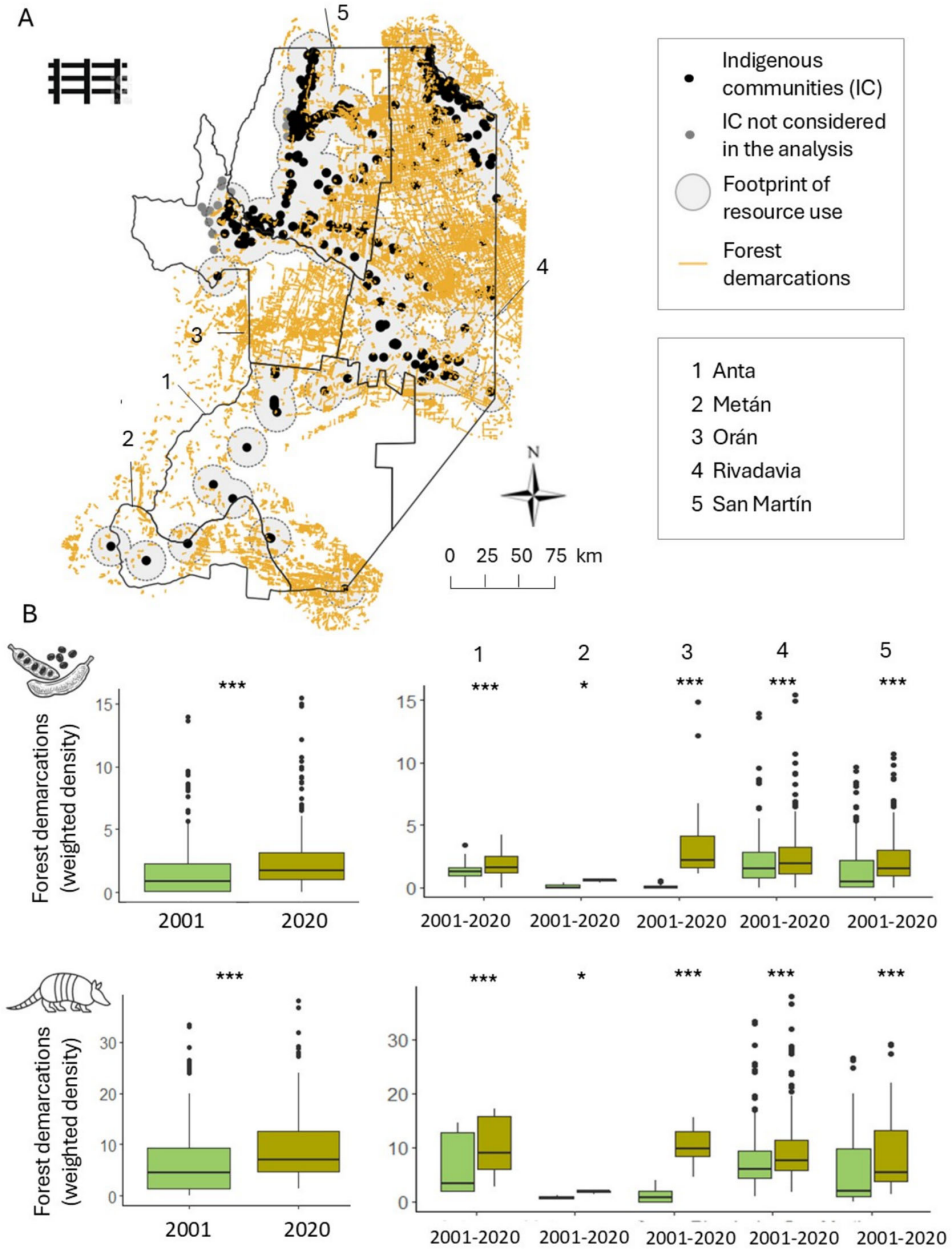
Access restrictions within the spatial footprints of forest use in 2001 and 2021. **A**. Map of forest demarcations in 2020; **B**. Weighted density of forest demarcations in the spatial footprints for plant-based (8 km buffer; top) and animal-based (15 km; bottom) resources in 2001 and 2021, for the whole study area (left) and by county (right). Significance levels: ***(*p* < 0.001); **(*p* < 0.01); *(*p* < 0.05); ns (not significant)

In 2020, the weighted density of forest demarcations—a proxy for access restrictions to forest resources—was, on average, 3.7 × higher within the spatial footprint of animal-based resources compared to that of plant-based resources (9.1 ± 0.6 vs 2.5 ± 0.2; Table S3). Within the spatial footprints of animal-based resources, Anta, Oran and Rivadavia had absolute values of demarcation density exceeding the overall average across all animal-based footprints by 16%, 11% and 6%. In contrast, San Martín and Metán recorded absolute values below the overall average, by 6 and 81%. Comparing the demarcation density across the spatial footprints of forest use between 2001 and 2020 revealed a significant increase in demarcation density, with an average rise of 41.6 ± 2.2%, and 174 communities (36% of the total) experienced demarcation density increases by more than 50%. For all counties and both resource groups, forest demarcations were significantly more extensive in 2020 than in 2001. We found the largest increases in forest demarcations in the footprints of forest use—on average for plant and animal-based resources—in Oran (90.1 ± 3.8%), followed by Metán (58.4 ± 33.9%), San Martín (53.3 ± 3.1%), Anta (30.6 ± 8.2%) and Rivadavia (19.1 ± 1.3%) (Fig. 5; Table S7 A–C).

**Fig. 6:**
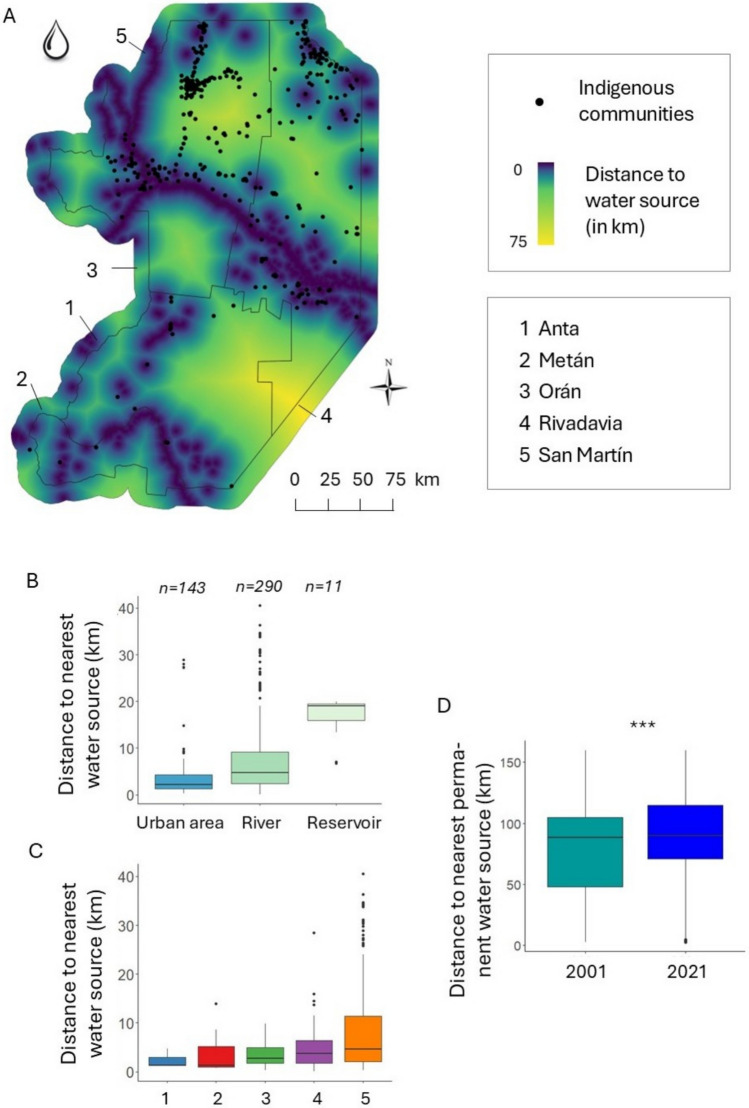
Access to water by Indigenous communities. **A**. Proximity map between Indigenous communities and permanent/seasonal water sources—including urban areas—in 2021; **B**. Nearest distance that the communities had to travel in 2021 to access a water source; **C**. Nearest distance that the communities had to travel in 2021 to access a water source by county; **D**. Nearest distance to a permanent natural water source in 2001 and 2021, excluding those in urban areas. Significance level: ***(*p* < 0.001). Differences between departments are presented in Figure S3

Regarding access to water, we found that by 2021, Indigenous communities of eastern Salta were, on average, located 6.6 ± 0.7 km away from a water source, including those in urban areas. Among these, 290 communities (65% of the total) had an urban area as their closest water source, at a mean distance of 3.8 ± 0.9 km. In contrast, 143 communities (32%) relied on a river as their nearest source, at a mean distance of 7.6 ± 0.9 km, while 11 communities (2%) had a reservoir as their closest source, at a mean distance of 16.5 ± 3.0 km. Alarmingly, 317 communities (71%) had to travel over 2 km to access water and 257 communities (58%) were located more than 10 km from a water source. This situation was particularly critical in San Martín and Rivadavia, where 73 (75%) and 112 (68%) communities, respectively, had to travel more than 2 km to access water. Similarly, 140 communities (61%) in San Martín and 90 communities (56%) in Rivadavia were located over 10 km from a water source (Fig. 6).

A comparison of the distances to permanent water bodies between 2001 and 2021 revealed a significant increase of 9.7 ± 3.0 km in the average distance Indigenous communities had to travel to access permanent natural water sources, excluding those in urban areas *(p* < *0.001)*. Notably, 39 communities (9% of the total) experienced substantial increases in travel distances to water—ranging from 92 to 122 km—while only 9 communities (2%) saw reductions of 2 to 7 km. When analysed by county, distances to permanent water bodies remained unchanged between 2001 and 2021 in San Martín and Orán. However, in Rivadavia, the average distance to a permanent water body increased significantly, by 28.0 ± 7.7 km (Fig. 6; Table S4; Table S8 A–C; Figure S3).

## Discussion

Deforestation poses a major risk to forest-dependent people, but the extent and spatial patterns of that risk generally remain elusive. This translates into barriers to developing and implementing policies, planning and enforcement to adequately protect these communities. By integrating participatory mapping with quantitative spatial analyses on remotely-sensed data, we mapped and quantified the impact of the expansion of industrialized agriculture on Indigenous communities in eastern Salta, Argentina. This region is part of the culturally diverse Dry Chaco region, a global deforestation hotspot hosting a wide range of Indigenous communities (Buchadas et al. 2022; Camino et al. 2023). Our analyses uncover how the advancing agricultural frontier, mainly driven by the expansion of industrial cropping and ranching, puts a considerable strain on forest-dependent Indigenous communities in three ways: (1) through a diminishing of forest area in the surrounding of Indigenous communities and an associated loss of ecosystem functioning, (2) through a substantial decrease in access to remaining forest around Indigenous communities, and (3) through limiting access to water sources. Collectively, these results make visible the stark consequences of agricultural expansion on forest-dependent Indigenous communities, and underscore both, the urgent need and potential for ‘putting forest-dependent people on the map’ to better consider them in sustainability planning in order to prevent their continued ecological marginalization in the Chaco and across other tropical dry woodlands.

Natural resource collection sites used by Indigenous communities within the forest matrix are not static but vary depending on the resource type, as shown by our results. They also change over time in response to various factors, including forest regeneration following resource extraction (e.g., plants and honey) and animal movements following hunting. Factors such as geography, resource availability, accessibility, seasonal fluctuations, competition with other actors, and community preferences all influence the distances travelled for resource collection. As a result, some communities may rely on larger or smaller areas depending on these factors. In our analysis, we defined buffer zones around each settlement by calculating the median collection distances by type of resource. For this, we used a large dataset of collection sites sampled by a group of communities to estimate the resource footprint of Indigenous communities in eastern Salta, capturing a wide variety of resource-use behaviours across communities with diverse ecological and cultural contexts. While this extrapolation is useful to understand the magnitude of resource use and how agricultural expansion impacts its spatial footprint, it does not fully capture the differences between communities or changes in resource-use footprints over time. To illustrate the latter, communities are increasingly using bicycles, motorcycles, and even vans to travel to collection sites, meaning that the footprints of use for some communities may have expanded over the past two decades to encompass areas with greater resource availability. Moreover, we highlight that the extrapolation process was not conducted in a participatory manner, so it might not fully account for potential cultural differences in the relationship of communities we extrapolated to with the forest.

The communities most affected by forest loss in their spatial footprints of forest use were those located in Anta (i.e., average forest area loss of 14.6 ± 5.9 and 18.5 ± 3.4% for the plant and animal-based resource footprints), which lies at the forefront of active deforestation frontiers. While intermediate forest loss was observed in San Martín (7.2 ± 1.1 and 9.3 ± 0.9%), the situation in that county was particularly concerning given that 52% of the communities identified in the study area were concentrated there. In contrast, Rivadavia, where 35% of the identified communities are located, had not yet seen the agricultural frontier advance to the same degree (0.9 ± 0.5 and 0.7 ± 0.9%), with potentially fewer conflicts in the region at the time of this study (Pratzer et al. 2025). Our study does not consider the agricultural expansion into other natural areas (e.g., natural grasslands) that can also impact Indigenous communities negatively. Generally though, the advance of large-scale agriculture in the province of Salta has led to widespread usurpations and evictions, as well as human and livestock intoxication and environmental contamination due to the use of agrochemicals (Redaf 2013; Schmidt 2021).

The observed decline in the Ecosystem Services Supply Index (ESSI) evidences a widespread deterioration in ecosystem functioning within the resource-use footprints, potentially affecting forest productivity, groundwater recharge, and biodiversity richness (Paruelo et al. 2016)—all aspects directly or indirectly related to the availability of plant and animal resources. Most communities in the study area experienced significant declines in ESSI between 2001 and 2021, with average reductions ranging from 13.6 ± 1.1% in Anta to 4.7 ± 0.3% in San Martín. In contrast, communities in Rivadavia showed a modest average increase of 1.6 ± 0.2% in ESSI, suggesting a relative improvement in resource availability, especially for those near the Pilcomayo and Bermejo rivers. However, it is crucial to note that forests in Rivadavia face harsher environmental conditions, reflected in the lower absolute ESSI values. This indicates that despite the relative increase we observed, the overall ecosystem capacity to support these communities remains limited. We note that our measure of ecosystem functionality, the ESSI, only proxies a set of quantifiable ecosystem services. In other words, it does not offer a full view on the diversity of forests’ contribution to the livelihoods of Indigenous communities. Integrating recognition of nature's contribution to people in future work, through valuation of the cultural services offered to people, for example (Díaz et al. 2018), would thus be an important extension of our work.

In addition to the deterioration of the forest-resource base through agricultural expansion, we uncovered a marked increase in restrictions in access for Indigenous communities to the remaining forests. This was evidenced by an average increase of 41.6 ± 2.2% in forest demarcations within the remaining forest matrix, in line with earlier work (del Giorgio et al. 2025). In 2020, Oran exhibited the highest weighted density of forest demarcations and a substantial average increase of 90.1 ± 3.8% in forest demarcations compared to 2001. Combined with the marked declines in the provisioning of ecosystem services in this county, these findings suggest that Oran faces an increasing challenge of limited resource availability and restricted access to natural resources provided by remaining forests. This loss of access to land and resources has profound economic, health, and nutritional consequences (Cernea 2005; Nerfa et al. 2020), with Indigenous communities increasingly relying on food purchased from local markets using social program funds or provided by government aid packages (Fundación Gran Chaco 2020). Restrictions in access have also fueled conflicts between smallholder groups, particularly between Indigenous communities and Creole (‘*Criollo*’) families—descendants of European immigrants who primarily engage in livestock farming. In part, the uptake of fencing by Creole families–done in an attempt to secure land claims in the face of advancing agro-industrial agriculture, as well as to facilitate livestock management in the changing landscape–has exacerbated tensions (Vallejos 2019).

Finally, our study highlights how the advance of the agricultural frontier not only restricts the availability of and access to forest-based resources but also leads to growing restrictions in terms of access to water. We found that communities were required to travel substantially longer average distances of 9.7 ± 3.0 km to reach a permanent water source for the studied period. It is important to highlight that, also here, our estimates are conservative. The distances we calculated were straight-line Euclidean distances, which do not account for roads, landscape features, or barriers, all of which mean that communities likely travel longer in reality. Moreover, the water accessibility issue is further compounded by the poor quality of the water available to Indigenous communities, which is a leading cause of infant mortality in the region (Jara 2022). In January 2020, a health emergency was declared in San Martín, Orán, and Rivadavia after the deaths of nine Wichí children, due in part to their inability to access drinking water (Provincial Law N° 8.185). In these counties, 75% of the rural population lacks access to safe drinking water (Fundación Gran Chaco 2020). While efforts to address this crisis have resulted in various water access projects —such as the installation of wells, water harvesting infrastructure, desalination units, and water transport via tanker trucks—, the problem remains largely unresolved. Several factors contribute to its persistence, including historical and structural water inequalities that are difficult to redress in the context of ongoing health and economic crises. Additionally, challenges related to the quality and maintenance of infrastructure, coupled with a lack of adaptation of water projects to the needs and practices of local communities, hinder an effective solution to the problem. The lack of community participation in the design and implementation of these projects often results in the target population failing to appropriately and effectively use new technologies, undermining the long-term success of these interventions (Schmidt and Tobias 2021).

## Conclusion

Many tropical dry forests worldwide are under high and rising pressure from expanding industrialized agriculture. As we uncover here for the Argentine Chaco, the impacts of these land-use changes on the resource base of forest-dependent people can be stark, and extend far beyond the (often already large) footprint of deforestation itself. Specifically, we highlight how the expansion of industrial agriculture diminishes the forest area around Indigenous communities and leads to a loss of ecosystem functioning in these areas. Moreover, where agriculture expands, Indigenous communities face a substantially decreasing access to remaining forests as well as to water sources. This highlights the importance of social and political institutions, and specifically land-use and sustainability planning carried out by them, to ensure the well-being of forest-dependent communities. Many of these communities do not have formal, recognized tenure. Recognizing and securing the land tenure of Indigenous Peoples is thus important to preserving ethnic diversity and alleviating poverty, while contributing to mitigate deforestation and environmental degradation (Reyes-García et al. 2022; Camino et al. 2023). Additionally, promoting collaborative governance is key, with efforts focused on creating incentives for marginalized groups to actively participate in decision-making processes (Cotroneo et al. 2021; Gadgil et al. 2021), including and involving Indigenous communities in forest management (Murali et al. 2022) and forest restoration (Aguiar et al. 2021). The evidence we provide here on the strong and detrimental impacts of agricultural expansion on Indigenous communities, as well as our approximation of the resource-use footprints of these communities, can inform sustainable development governance, such as incorporating the needs of Indigenous communities in Argentina’s land-use planning processes (e.g., National Law No. 23331 of “Minimum Standards for the Environmental Protection of Native Forests”). Despite numerical valuation based on satellite imagery and spatial processing is limited in understanding the magnitude and complexity of the impact, we hope our approach can help shed light on the barriers to resource access faced by Indigenous communities, while advocating for their inclusion in policy discussions, planning processes and legal claims generated by conflicting land uses.

## Supplementary Information

Below is the link to the electronic supplementary material.

Supplementary file1 (PDF 483 kb)

## Footnotes

1 http://monitoreodesmontes.com.ar.
