# Supplementary material for: Impacts of agricultural expansion on the resource availability of forest-dependent Indigenous communities in the Dry Chaco
Source: Ambio. 2025 Jul 31;55(1):147–63. doi: 10.1007/s13280-025-02217-6 (PMC12672981; doi:10.1007/s13280-025-02217-6)
Supplement: Supplementary file 1 — Supplementary file1 (PDF 483 kb) [file 13280_2025_2217_MOESM1_ESM.pdf]

Ambio

Supplementary Information

This supplementary information has not been peer reviewed

Title: **Impacts of agricultural expansion on the resource availability of forest-dependent Indigenous communities in the Dry Chaco**

Supplementary Information 1: Agricultural expansion in eastern Salta

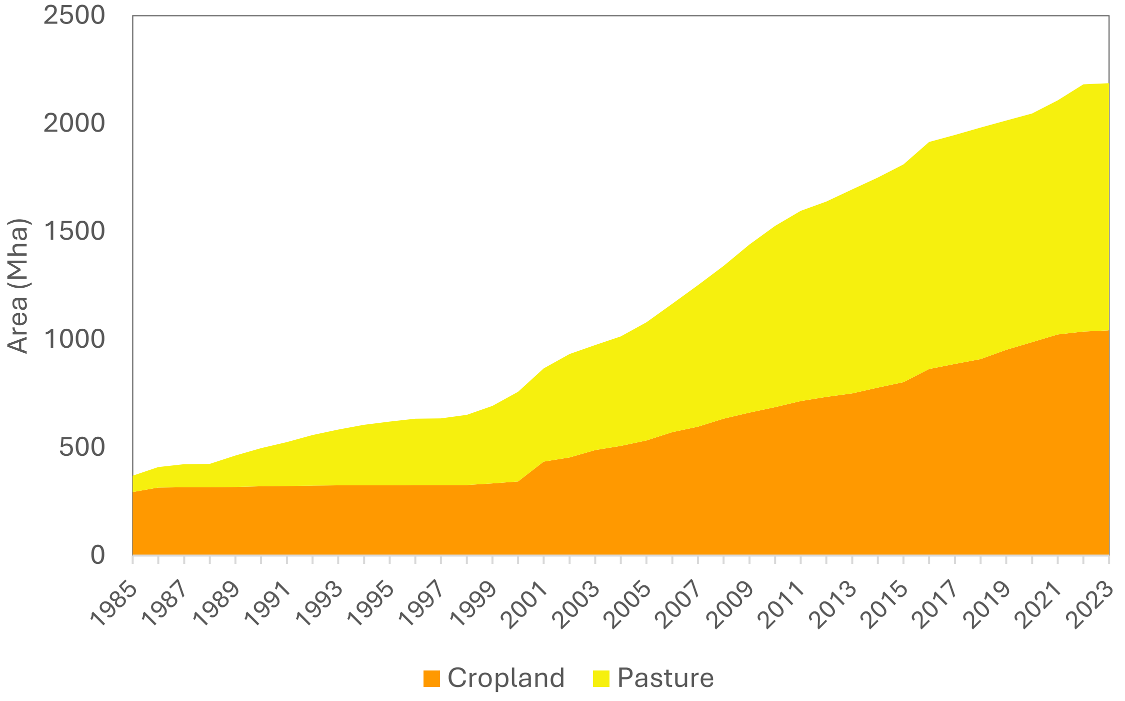

Figure S1. Cumulative area of cropland and pastures over time in eastern Salta, from 1985 to 2023.

Supplementary Information 2: Distances travelled by activity

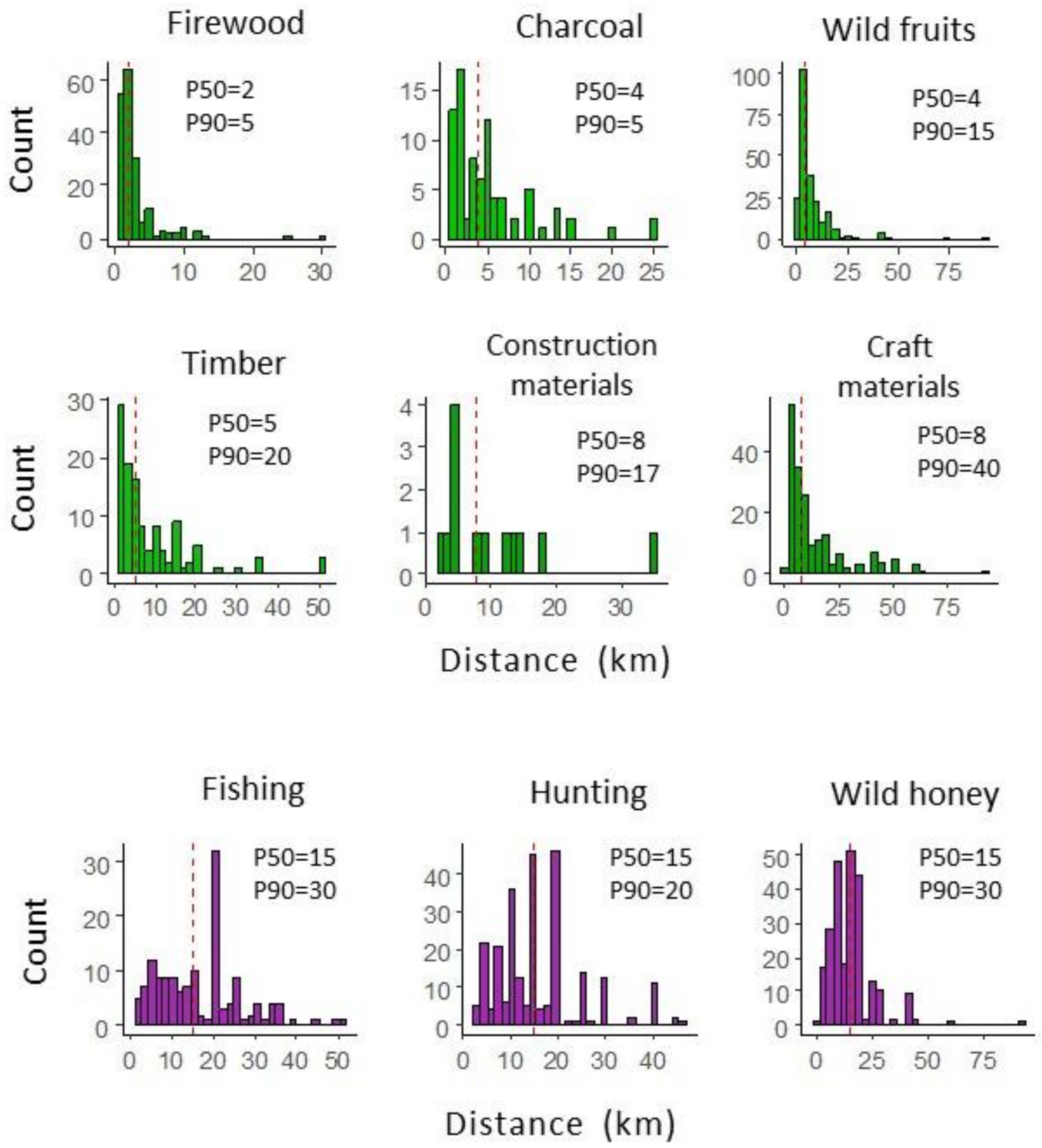

Figure S2. Histograms of distances by activity. The red dotted line marks the median distance.

Supplementary Information 3: Absolute mean values

Table S1. Absolute Forest loss within footprints *(ha)*

Supplementary Table 1A. Absolute Forest loss: 2001 and 2021 (ha)

	2001		2021	
	**Forest loss mean**	**Forest loss Std Error**	**Forest loss mean**	**Forest loss Std Error**
**08km**	**3324,6**	**184,5**	**4375,9**	**218,9**
Anta	7632,7	1113,7	10523,3	1056,2
Metan	6811,7	3361,4	7060,2	3485,6
Oran	8018,5	579,7	9536,4	527,9
Rivadavia	50,2	16,5	233,2	59,8
San Martin	4425,0	224,9	5851,1	263,9
**15km**	**10337,0**	**540,4**	**14847,6**	**696,9**
Anta	24349,4	3008,6	37217,7	3201,7
Metan	21570,7	10785,4	22429,9	11070,4
Oran	26972,9	1511,9	32204,0	1537,6
Rivadavia	105,0	25,8	589,1	132,1
San Martin	13427,9	591,3	19908,5	753,7
**Grand Total**	**6830,8**	**308,7**	**9611,7**	**405,1**

Supplementary Table 1B. Changes in absolute Forest loss between 2001 and 2021 (ha)

	Mean forest loss	Std Error
**08 km**	**1051,3**	**75,0**
Anta	2890,7	598,5
Metan	248,5	124,3
Oran	1517,8	180,1
Rivadavia	183,0	47,3
San Martin	1426,1	112,0
**15 km**	**4510,6**	**231,9**
Anta	12868,3	1202,1
Metan	859,2	285,1
Oran	5231,1	283,5
Rivadavia	484,1	113,8
San Martin	6480,6	302,6
**Grand Total**	**2780,9**	**134,9**

Table S2. Ecosystem Services Supply Index *(ESSI - relative values 0 to 1)*

	2001		2021	
	**ESSI mean**	**ESSI Std Error**	**ESSI mean**	**ESSI Std Error**
**08km**	**0,660**	0,002	**0,635**	0,003
Anta	0,660	0,008	0,554	0,011
Metan	0,688	0,073	0,654	0,050
Oran	0,723	0,007	0,662	0,010
Rivadavia	0,636	0,002	0,612	0,003
San Martin	0,666	0,003	0,653	0,005
**15km**	**0,668**	0,002	**0,639**	0,003
Anta	0,675	0,005	0,546	0,009
Metan	0,715	0,066	0,693	0,050
Oran	0,734	0,006	0,672	0,010
Rivadavia	0,641	0,002	0,616	0,002
San Martin	0,675	0,002	0,657	0,004
**Grand Total**	**0,664**	0,001	**0,637**	0,002

Table S3. Forest Demarcations Weighted Density (FDWD, sum of demarcation pixels weighted by the amount of forest within each buffer)

	2001		2020	
	**FDWD mean**	**FDWD Std Error**	**FDWD mean**	**FDWD Std Error**
**08km**	**1,63**	**0,10**	**2,47**	**0,11**
Anta	1,34	0,19	1,81	0,26
Metan	0,13	0,13	0,57	0,08
Oran	0,10	0,04	3,49	0,65
Rivadavia	2,16	0,18	2,66	0,20
San Martin	1,49	0,14	2,30	0,14
**15km**	**6,24**	**0,30**	**9,10**	**0,31**
Anta	6,54	1,24	10,52	1,25
Metan	0,82	0,19	1,76	0,18
Oran	1,16	0,28	10,14	0,68
Rivadavia	7,91	0,48	9,67	0,55
San Martin	5,58	0,43	8,52	0,43
**Grand Total**	**3,86**	**0,17**	**5,68**	**0,20**

Table S4. Distance to nearest water source *(km)*

	2001		202	
	**Distance mean**	**Distance Std Error**	**Distance mean**	**Distance Std Error**
Anta	37,36	2,80	35,98	2,78
Metan	71,13	0,84	69,19	0,85
Oran	96,86	10,96	96,86	10,96
Rivadavia	78,81	23,88	106,85	29,09
San Martin	77,72	40,89	77,72	40,89
**Grand Total**	**77,84**	**73,11**	**87,62**	**75,12**

Supplementary Information 4: Statistical Tests

Forest Loss

Table S5 A. Forest loss: Normality test

p-value	08 km		15 km	
	**2001**	**2021**	**2001**	**2021**
TOTAL *(n=444)*	2.899718e-22 ***	1.864938e-20 ***	2.899718e-22 ***	1.864938e-20 ***
1.Anta *(n=19)*	0.02407695 *	0.01100535 *	0.138672	0.01600997 *
2.Metán *(n=3)*	0.000951314***	0.0009332201***	0.0006083078 ***	0.0005677436 ***
3.Orán *(n=35)*	0.2308075	0.05237328	0.003837733 **	0.003406432**
4.Rivadavia *(n=156)*	8.464797e-25 ***	2.191938e-23 ***	3.705369e-23 ***	1.515157e-22 ***
5.San Martín *(n=231)*	2.985568e-09 ***	1.337807e-07 ***	2.985568e-09 ***	1.337807e-07 ***

Shapiro-Wilk test (p values). *Significant differences: **** (*p<0.001*); ** (*p<0.01*); * (*p<0.05*).

p-value > 0.05 → Data is likely normal (fail to reject normality).

p-value ≤ 0.05 → Data is not normal (reject normality) -> used Wilcoxon Signed-Rank Test

Table S5 B. Forest loss: Homogeneity of Variances

p-value	**08 km**	**15 km**
TOTAL *(n=444)*	2.532e-05 ***	6.266e-09 ***
1.Anta *(n=19)*	0.7591	0.99
2.Metán *(n=3)*	0.9807	0.9862
3.Orán *(n=35)*	0.3898	0.8195
4.Rivadavia *(n=156)*	0.003444 **	0.0003703 ***
5.San Martín *(n=231)*	0.0202 *	0.005895 **

Levene Test (p-values). *Significant differences: **** (*p<0.001*); ** (*p<0.01*); * (*p<0.05*).

p-value > 0.05 → Variances are homogeneous (equal).

p-value ≤ 0.05 → Variances are not equal -> used Welch's t-test instead.

Table S5 C. Forest loss: Paired differences

p-value	**08 km**	**15 km**
TOTAL *(n=444)*	< 2.2e-16 ***	< 2.2e-16 ***
1.Anta *(n=19)*	3.815e-06 ***	3.815e-06 ***
2.Metán *(n=3)*	0.3711	0.25
3.Orán *(n=35)*	7.7e-10 ***	5.821e-11 ***
4.Rivadavia *(n=156)*	7.678e-12 ***	< 2.2e-16 ***
5.San Martín *(n=231)*	< 2.2e-16 ***	< 2.2e-16 ***

Wilcoxon Signed-Rank Test (if not normal distribution), or Paired t-test (if normal distribution). *NOTE*: Paired t-tests (if normal distribution) or Wilcoxon Signed-Rank tests (if not normal distribution) are still robust for big sample size (n>30), even if variances are not homogeneous. *Significant differences: *** (p<0.001); ** (p<0.01); * (p<0.05).*

p-value > 0.05 → Mean difference is equal to 0.

p-value ≤ 0.05 → Mean difference is not equal to 0.

Ecosystem Services Supply Index (ESSI)

Table S6 A. ESSI: Normality test

p-value	08 km		15 km	
	**2001**	**2021**	**2001**	**2021**
TOTAL *(n=444)*	3.747938e-09	2.408217e-11	3.747938e-09	2.408217e-11
1.Anta *(n=19)*	0.3562713	0.3215212	9.436844e-05 ***	0.02049083*
2.Metán *(n=3)*	0.0004992191***	0.0009921183***	8.186776e-05***	2.091923e-05***
3.Orán *(n=35)*	0.003390418**	4.967241e-05***	0.01146405 *	0.001558563**
4.Rivadavia *(n=156)*	6.879178e-07***	0.001080693**	0.0001903388***	0.008655108**
5.San Martín *(n=231)*	7.040966e-06***	7.574351e-06 ***	7.040966e-06 ***	7.574351e-06***

Shapiro-Wilk test (p values). *Significant differences: **** (*p<0.001*); ** (*p<0.01*); * (*p<0.05*).

p-value > 0.05 → Data is likely normal (fail to reject normality).

p-value ≤ 0.05 → Data is not normal (reject normality)-> used Wilcoxon Signed-Rank Test.

Table S6 B. ESSI: Homogeneity of Variances

p-value	**08 km**	**15 km**
TOTAL *(n=444)*	1.419e-07 ***	3.827e-12 ***
1.Anta *(n=19)*	0.125	0.005268 **
2.Metán *(n=3)*	0.8134	0.8548
3.Orán *(n=35)*	0.8435	0.007576 **
4.Rivadavia *(n=156)*	0.00274 **	0.0005585 ***
5.San Martín *(n=231)*	8.025e-11 ***	< 2.2e-16 ***

Levene Test (p-values). *Significant differences: **** (*p<0.001*); ** (*p<0.01*); * (*p<0.05*).

p-value > 0.05 → Variances are homogeneous (equal).

p-value ≤ 0.05 → Variances are not equal (used Welch's t-test instead).

Table S6 C. ESSI: Paired differences

p-value	**08 km**	**15 km**
TOTAL *(n=444)*	< 2.2e-16 ***	< 2.2e-16 ***
1.Anta *(n=19)*	1.388e-07***	3.815e-06***
2.Metán *(n=3)*	0.5*	0.5*
3.Orán *(n=35)*	8.149e-10***	7.974e-09***
4.Rivadavia *(n=156)*	9.262e-15***	< 2.2e-16***
5.San Martín *(n=231)*	0.0002611***	0.0002611***

Wilcoxon Signed-Rank Test (if not normal distribution), or Paired t-test (if normal distribution).

NOTE: Paired t-tests (if normal distribution) or Wilcoxon Signed-Rank tests (if not normal distribution) are still robust for big sample sizes (n>30), even if variances are not homogeneous.

*Significant differences: *** (p<0.001); ** (p<0.01); * (p<0.05).*

p-value > 0.05 → Mean difference is equal to 0.

p-value ≤ 0.05 → Mean difference is not equal to 0.

Weighted Density of Forest Demarcations (WDFD)

Table S7 A. WDFD: Normality test

p-value	08 km		15 km	
	**2001**	**2020**	**2001**	**2021**
TOTAL *(n=444)*	1.531686e-24 ***	1.132541e-22 ***	1.531686e-24 ***	1.132541e-22 ***
1.Anta *(n=19)*	0.2076409	0.4485719	0.0002721726 ***	0.01574035 *
2.Metán *(n=3)*	0 ***	0.006737189 **	0 ***	0.003065774 **
3.Orán *(n=35)*	1.932776e-07 ***	2.296042e-06 ***	0.0004050979 ***	0.4690697
4.Rivadavia *(n=156)*	9.136753e-15 ***	1.860755e-13 ***	2.59068e-13 ***	2.402206e-12 ***
5.San Martín *(n=231)*	2.974865e-18 ***	7.953912e-15 ***	2.974865e-18 ***	7.953912e-15 ***

Shapiro-Wilk test (p values). *Significant differences: **** (*p<0.001*); ** (*p<0.01*); * (*p<0.05*).

p-value > 0.05 → Data is likely normal (fail to reject normality).

p-value ≤ 0.05 → Data is not normal (reject normality)-> used Wilcoxon Signed-Rank Test

Table S7 B. WDFD: Homogeneity of Variances

p-value	**08 km**	**15 km**
TOTAL *(n=444)*	0.2394	0.5629
1.Anta *(n=19)*	0.2386	0.6563
2.Metán *(n=3)*	0.7558	0.9732
3.Orán *(n=35)*	0.002522 **	0.001151 **
4.Rivadavia *(n=156)*	0.3022	0.2765
5.San Martín *(n=231)*	0.6831	0.8184

Levene Test (p-values). *Significant differences: **** (*p<0.001*); ** (*p<0.01*); * (*p<0.05*).

p-value > 0.05 → Variances are homogeneous (equal).

p-value ≤ 0.05 → Variances are not equal -> used Welch's t-test instead.

Table S7 C. WDFD: Paired differences

p-value	**08 km**	**15 km**
TOTAL *(n=444)*	< 2.2e-16 ***	< 2.2e-16 ***
1.Anta *(n=19)*	0.0136 *	3.815e-06
2.Metán *(n=3)*	0.3711	0.25
3.Orán *(n=35)*	8.796e-06 ***	2.384e-07 ***
4.Rivadavia *(n=156)*	< 2.2e-16 ***	< 2.2e-16 ***
5.San Martín *(n=231)*	< 2.2e-16 ***	< 2.2e-16 ***

Wilcoxon Signed-Rank Test (if not normal distribution), or Paired t-test (if normal distribution).

NOTE: Paired t-tests (if normal distribution) or Wilcoxon Signed-Rank tests (if not normal distribution) are still robust for big sample sizes (n>30), even if variances are not homogeneous.

*Significant differences: *** (p<0.001); ** (p<0.01); * (p<0.05).*

p-value > 0.05 → Mean difference is equal to 0.

p-value ≤ 0.05 → Mean difference is not equal to 0.

Distance to water sources

Table S8 A. Distance to water sources: Normality test

p-value	**2001**	**2020**
TOTAL *(n=444)*	2.541722e-12 ***	1.951354e-11 ***
1.Anta *(n=19)*	4.334199e-05 ***	3.217512e-05 ***
2.Metán *(n=3)*	9.564034e-05 ***	0.0001142436 ***
3.Orán *(n=35)*	0.07785311	0.07785311
4.Rivadavia *(n=156)*	1.022078e-09 ***	4.94749e-09 ***
5.San Martín *(n=231)*	6.063435e-08 ***	6.063435e-08 ***

Shapiro-Wilk test (p-values). *Significant differences: **** (*p<0.001*); ** (*p<0.01*); * (*p<0.05*).

p-value > 0.05 → Data is likely normal (fail to reject normality).

p-value ≤ 0.05 → Data is not normal (reject normality) -> used Wilcoxon Signed-Rank Test

Table S8 B. Distance to water sources: Homogeneity of Variances

	p-value
TOTAL *(n=444)*	0.1359
1.Anta *(n=19)*	0.9786
2.Metán *(n=3)*	0.9895
3.Orán *(n=35)*	1
4.Rivadavia *(n=156)*	6.985e-09 ***
5.San Martín *(n=231)*	1

Levene Test (p-values). *Significant differences: **** (*p<0.001*); ** (*p<0.01*); * (*p<0.05*).

p-value > 0.05 → Variances are homogeneous (equal).

p-value ≤ 0.05 → Variances are not equal -> used Welch's t-test instead.

Table S8 C. Distance to water sources: Paired differences

TOTAL *(n=444)*	2.634e-08 ***
1.Anta *(n=19)*	0.03603 *
2.Metán *(n=3)*	0.25
3.Orán *(n=35)*	*no difference*
4.Rivadavia *(n=156)*	5.465e-08 ***
5.San Martín *(n=231)*	*no difference*

Wilcoxon Signed-Rank Test (if not normal distribution), or Paired t-test (if normal distribution).

NOTE: Paired t-tests (if normal distribution) or Wilcoxon Signed-Rank tests (if not normal distribution) are still robust for big sample sizes (n>30), even if variances are not homogeneous.

*Significant differences: *** (p<0.001); ** (p<0.01); * (p<0.05).*

p-value > 0.05 → Mean difference is equal to 0.

p-value ≤ 0.05 → Mean difference is not equal to 0.

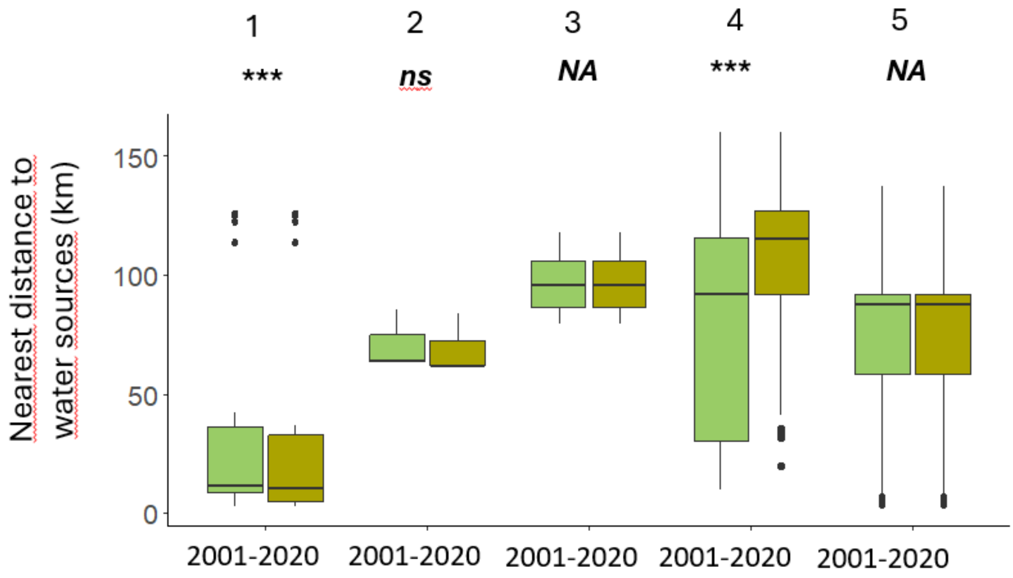

Refs: 1. Anta, 2. Metán, 3. Orán, 4. Rivadavia, 5. San Martín

*Significant differences: **** (*p<0.001*); ** (*p<0.01*); * (*p<0.05*), ns: not significant

Figure S3. Significant paired differences between departments
